# A case of exaggerated exuberance: Iatrogenic atrioventricular block/intra‐Hisian Wenckebach during conduction system pacing

**DOI:** 10.1002/joa3.12968

**Published:** 2023-12-13

**Authors:** Anindya Ghosh, Anbarasan Sekar, Chenni S. Sriram, Ulhas M. Pandurangi

**Affiliations:** ^1^ Department of Cardiac Electrophysiology and Pacing, Arrhythmia Heart Failure Academy The Madras Medical Mission Chennai India; ^2^ Division of Cardiology, Sub‐Section of Electrophysiology Children's Hospital of Michigan and Detroit Medical Center Detroit Michigan USA

**Keywords:** AV Wenckebach, conduction system pacing, iatrogenic, intra‐Hisian, sinus node dysfunction

## Abstract

Isolated sinus node dysfunction with its pursuant long‐term risk for atrioventricular (AV) conduction disease poses a unique dilemma for proponents of CSP due to paucity of imprimatur guidelines. In such scenarios, the risk and prognosis of iatrogenic AV block is not well elucidated but is a valid concern. We report a case where CSP was complicated by iatrogenic AV block and peculiarly the rare phenomenon of intra‐Hisian Wenckebach.

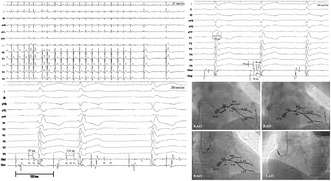

Conduction system pacing (CSP) is increasingly employed for physiological cardiac resynchronization. In many centers including ours, it is exuberantly utilized as the de facto pacing strategy. Isolated sinus node dysfunction with its pursuant long‐term risk for atrioventricular (AV) conduction disease poses a unique dilemma for proponents of CSP due to paucity of imprimatur guidelines. In such scenarios, the risk and prognosis of iatrogenic AV block is not well elucidated but is a valid concern. We report such a case where CSP was complicated by iatrogenic AV block and peculiarly the rare phenomenon of intra‐Hisian Wenckebach.

A 74‐year‐old gentleman with a structurally normal heart, normal atrioventricular (AV) conduction, and narrow QRS was diagnosed with symptomatic sinus node dysfunction.

His symptoms correlated with sinus pauses noted during ambulatory electrocardiogram (ECG) monitoring. Conduction system pacing (CSP) was planned as per our default institutional protocol. Dual chamber transvenous permanent pacemaker implantation was then performed targeting the proximal left bundle branch area (LBBa). A lumenless ventricular lead (Medtronic) guided by a fixed dual curve delivery sheath (C315His, Medtronic Inc.) was utilized for this purpose.

The delivery sheath housing the retracted lead was maneuvered over the right ventricular (RV) endocardial aspect of the proximal AV septum as a prelude to target the proximal LBBa (Video S1 and Figure 1A). Coincident to this, inadvertent injury to the AV conduction system was noted (Figure 1A). There was a transient right bundle branch block (RBBB) followed by a complete AV block (CAVB). The latter was associated with a bradycardic narrow QRS junctional escape rhythm (30–35 bpm). Subsequently, backup temporary RV pacing was initiated.

**FIGURE 1:**
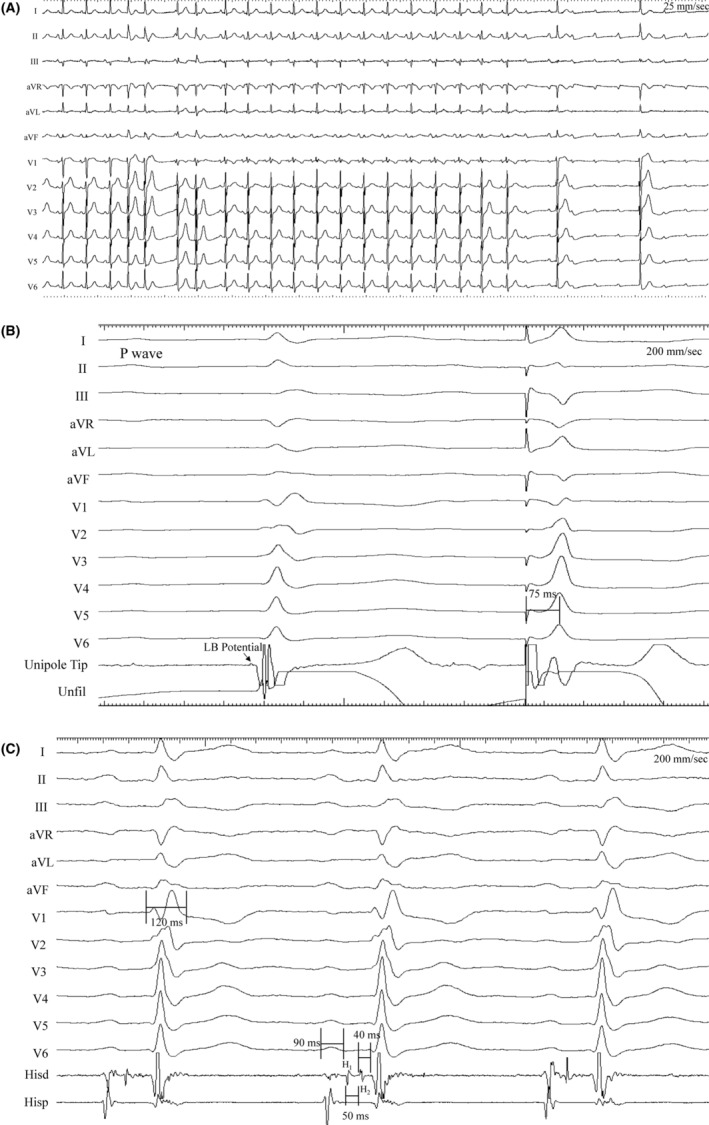
(A) 12‐lead electrocardiogram (ECG) while maneuvering the delivery catheter toward the proximal atrioventricular (AV) septum for left bundle branch area pacing (see text for details). (B) 12‐lead ECG with intracardiac EGMs from the lead tip in unipolar configuration during intrinsic rhythm and pacing at the corresponding site after lead deployment. Note the local potential labeled as a left bundle (LB) potential. The paced QRS morphology shows an incomplete right bundle branch block (RBBB) pattern with Qr in V1, QRSd of 110 ms, and left ventricular activation time (LVAT) of 75 ms. (C) 12‐lead ECG with intracardiac EGMs from a quadripolar catheter placed at the His bundle region. Note the prolonged PR interval, split His potentials, and normal H2‐V interval (see text for details).

The permanent pacing lead was then deployed through the sheath and screwed in after adjudicating the appropriate target site based on previously established criteria.^1^ By this time, there was resolution of CAVB albeit with residual first‐degree AV block/RBBB and intermittent AV Wenckebach. During AV conduction, the unipolar tip electrograms obtained from the pacing lead demonstrated an LB potential (Figure 1B). LBBa capture was ascertained during pacing as demonstrated by, (i) an incomplete RBBB pattern (Qr in V1), (ii) QRS duration of 100 ms, and (iii) fixed left ventricular activation time (LVAT) of 75 ms (Figure 1B).^1^

A quadripolar catheter was then placed over the His bundle area to analyze the characteristics/level of AV block. During sinus rhythm (cycle length or CL of 785 ms), there was 1:1 AV conduction with RBBB along with a split His bundle potential (H1‐H2 interval of 50 ms; Figure 1C). The distal His to V (H2‐V) was normal (40 ms). Intra‐Hisian Wenckebach was demonstrated during pacing at a faster CL of 520 ms (Figure 2). There is a progressive prolongation of the H1‐H2 interval culminating with an intra‐Hisian block (H1‐but no H2).

**FIGURE 2:**
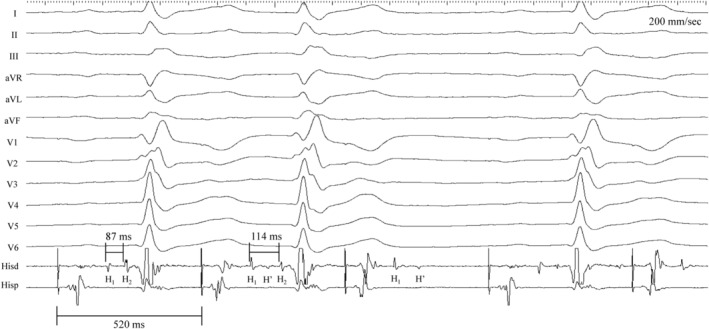
12‐lead ECG with intracardiac EGMs during atrial pacing at a cycle length of 520 ms. Note the presence of 3:2 AV Wenckebach with block at intra‐Hisian level (see text for details).

Because of this, the patient required the pacemaker to be programmed to a DDDR mode (Lower rate limit of 70 bpm, Upper rate limit of 130 bpm) with a nominal AV delay. The postimplant 12‐lead ECG showed optimal evidence of CSP (Figure 3). At the time of discharge (3 days postimplant), there was 1:1 AV conduction during atrial pacing up to 130 bpm, although with residual first‐degree AV block/RBBB.

**FIGURE 3:**
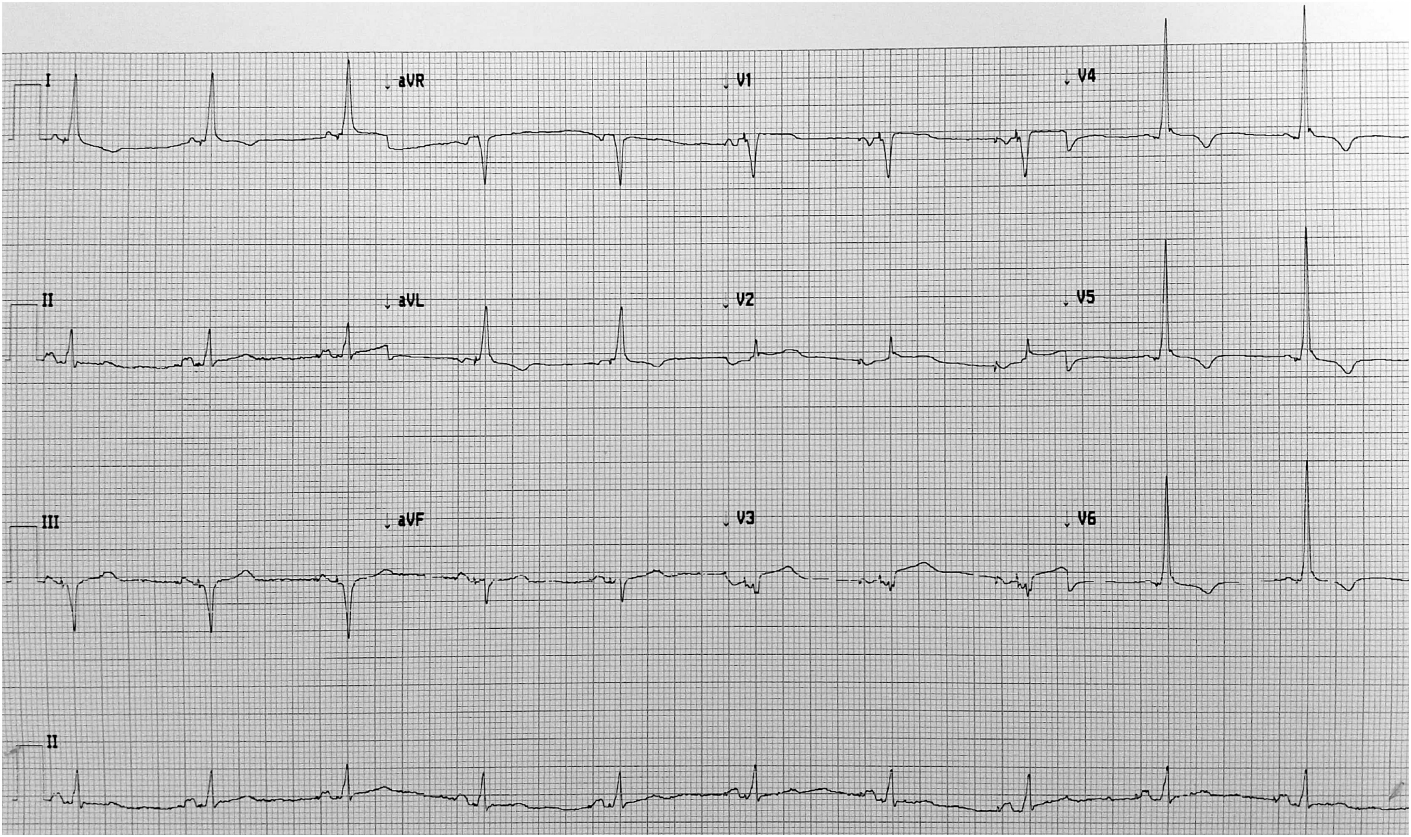
12‐lead ECG in DDDR mode on postimplant day 1.

However, follow‐up interrogation at 1 month in AAI mode revealed sinus rhythm with 1:1 AV conduction, normal PR interval (140 ms), and a narrow QRS with resolution of RBBB (Figure S2).

The present case highlights some important caveats when implementing the CSP strategy in patients with intact AV conduction. The risk of iatrogenic AV block as shown here is a valid concern, perhaps more so than with preexisting AV conduction disease. Its incidence/prognosis is a nascent area of research.^2^ The compactness of proximal conduction system (His bundle/proximal LBBa) may predispose it to a heightened risk of hardware‐induced trauma. Mechanical trauma is posited at the level of His bundle even though the eventual target for CSP was the proximal LBBa. This is the most likely etiology of the reported intra‐Hisian Wenckebach. It is anticipated that Figure S1 would render a feasible insight into this phenomenon. During proximal LBBaP, lead manipulation coupled with cardiac motion may result in inadvertent/iatrogenic trauma to the bundle of His at the level of membranous septum. Here the His bundle penetrates the gap between the septal and anterior leaflets of the tricuspid valve to lie on the crest of the ventricular septum prior to its bifurcation. Presently, the distal LBBa has emerged as a more common and larger target area for CSP.^1^ It theoretically posits a lower risk for iatrogenic AV block, but this supposition is bereft of empirical data.

The etiology of AV Wenckebach in such scenarios is not to be automatically inferred as of AV nodal origin. It should be meticulously investigated as showcased here in the example of intra‐Hisian Wenckebach. In addition, the presence of RBBB during 1:1 AV conduction followed by normalization of the QRS during junctional escape rhythm associated with CAVB (Figure 1A) merits a brief explanation. It is likely that there was iatrogenic injury to both the His/Right bundle. The latter manifested as a rate‐/acceleration‐dependent block with a resolution during the junctional escape bradycardia.

In the context of isolated sinus node dysfunction, the role of CSP may itself be questionable. The long‐term risk of ventricular pacing requiring reoperation (need to change pacing mode from AAIR) in such patients is estimated to be around 9%.^3^ Currently, CSP has garnered strong guideline‐based recommendations (class of recommendation 2a or above) only when significant ventricular pacing is anticipated at the outset.^4^ A less robust class 2b endorsement is sanctioned for LBBaP even when less than substantial ventricular pacing is anticipated.^5^ Therefore, the risks versus benefits of CSP vis‐à‐vis RV endocardial pacing warrants thorough deliberation when balancing any future anticipation of ventricular pacing. The authors hope that any such limited reports will generate focused research yielding a more data‐driven approach to CSP for isolated sinus node dysfunction.

## CONFLICT OF INTEREST STATEMENT

Authors declare no conflict of interests for this article.

## Supporting information


Video S1.

Figures S1–S2.

Captions.

## Data Availability

The data that support the findings of this study are available from the corresponding author upon reasonable request.
