# Supplementary material for: A case of exaggerated exuberance: Iatrogenic atrioventricular block/intra‐Hisian Wenckebach during conduction system pacing
Source: J Arrhythm. 2023 Dec 13;40(1):156–9. doi: 10.1002/joa3.12968 (PMC10848609; doi:10.1002/joa3.12968)
Supplement: Supplementary file 2 — Figures S1–S2. [file JOA3-40-156-s002.pptx]

## Slide 1
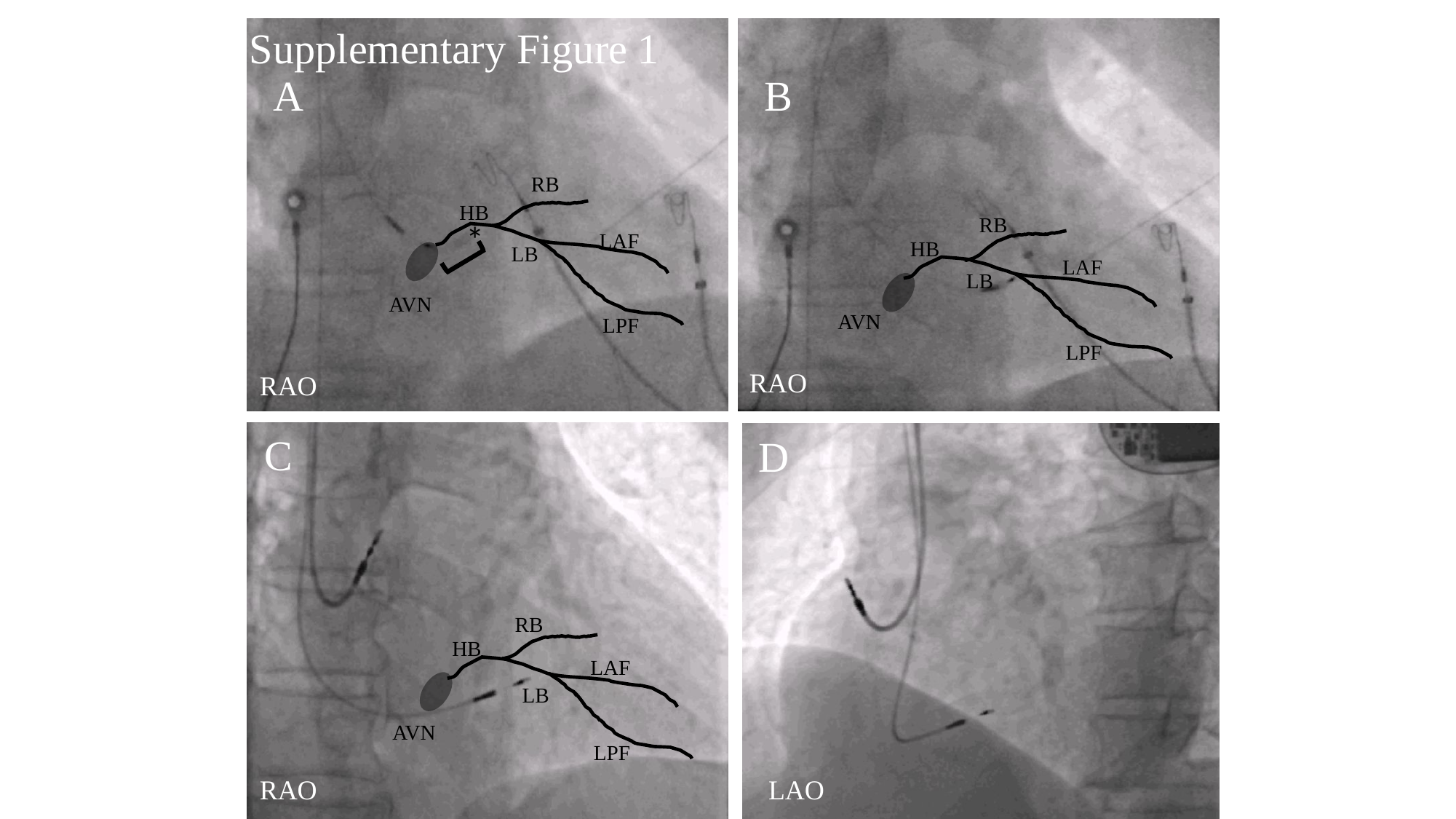

Supplementary Figure 1
RB
HB
LAF
LB
LPF
A
B
RB
HB
LAF
LB
LPF
*
AVN
AVN
RAO
RAO
C
D
RB
HB
LAF
LB
LPF
AVN
RAO
LAO

## Slide 2
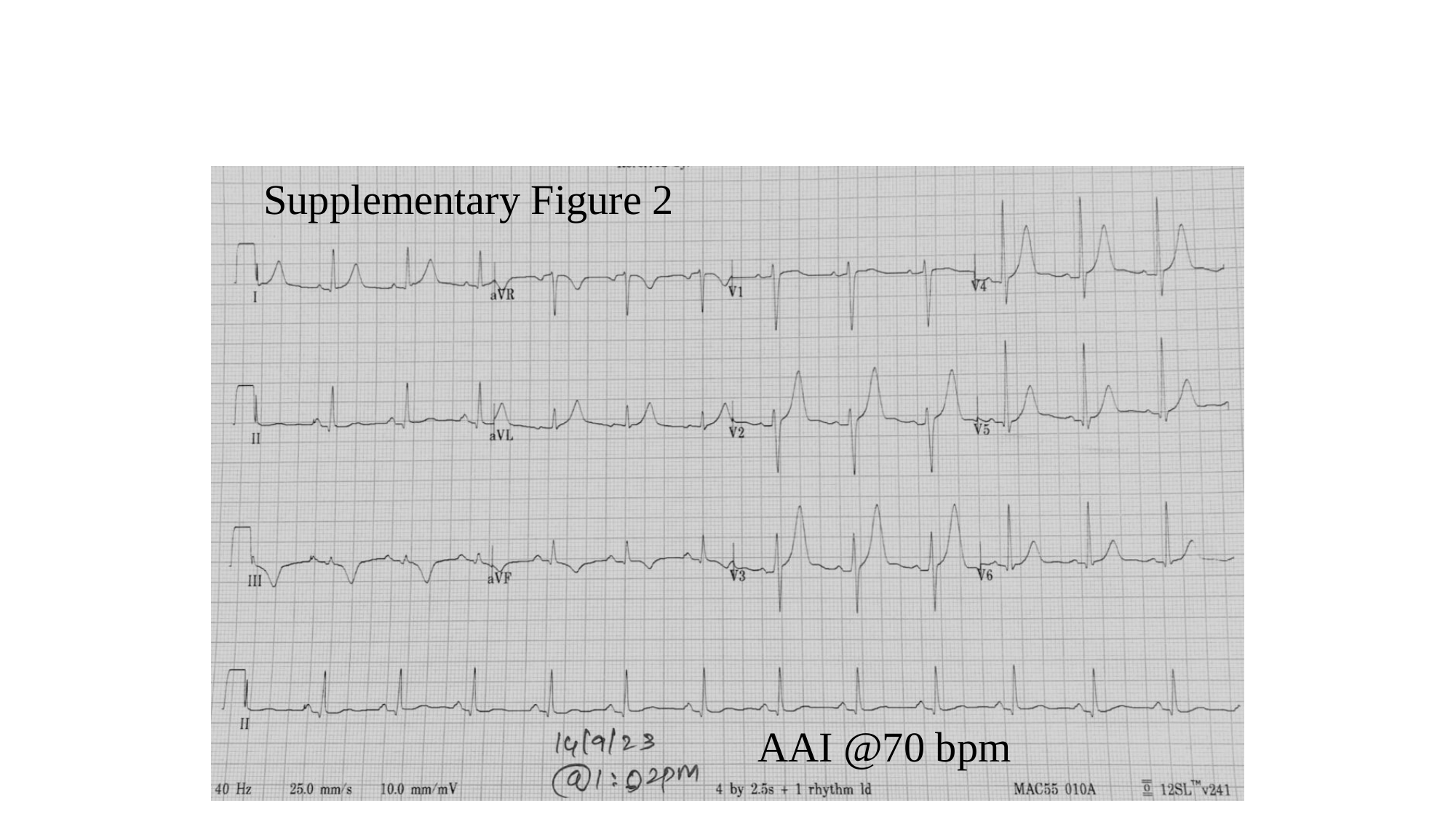

Supplementary Figure 2
AAI @70 bpm
