# Supplementary material for: A case of exaggerated exuberance: Iatrogenic atrioventricular block/intra‐Hisian Wenckebach during conduction system pacing
Source: J Arrhythm. 2023 Dec 13;40(1):156–9. doi: 10.1002/joa3.12968 (PMC10848609; doi:10.1002/joa3.12968)
Supplement: Supplementary file 3 — Captions. [file JOA3-40-156-s001.docx]

Video S1. Delivery catheter manipulation leading to AV conduction system injury.

Figure S1 Fluoroscopic views of delivery catheter position (A, B) during iatrogenic atrioventricular conduction injury with superimposed schema of putative conduction system anatomy and final lead position (C, D) in orthogonal planes. 1A depicts the orientation of the sheath () and its tip (*) at the level of the penetrating His bundle (postulated site of mechanical trauma).

Figure S2 12-lead ECG with intracardiac in AAI mode at 1-month follow-up showing resolution of first-degree AV block and RBBB with narrow QRS.
